# One health approach to mitigate anthrax in Ghana

**DOI:** 10.1002/hsr2.1807

**Published:** 2024-01-09

**Authors:** Malik O. Oduoye, Godfred Y. Scott, Tirth Dave, Akanbi‐Hakeem H. Bolanle, Alexandra D. Mwinbong, Olajide O. Modupeoluwa

**Affiliations:** ^1^ Department of Research Medical Research Circle (MedReC) Bukavu Democratic Republic of the Congo; ^2^ Department of Medical Diagnostics Kwame Nkrumah University of Science and Technology Kumasi Ghana; ^3^ Bukovinian State Medical University Chernivtsi Ukraine; ^4^ Department of Community Medicine Nile University of Nigeria Abuja Nigeria; ^5^ Department of Biotechnology University for Development Studies Tamale Ghana; ^6^ Department of Public Health National Open University of Nigeria Abuja Nigeria

**Keywords:** anthrax, *Bacillus anthracis*, Ghana, One Health, public health, zoonotic disease

## Abstract

Anthrax outbreaks in Ghana have become a pressing public health concern, posing threats to human health, the agricultural sector, and social well‐being. This letter to the editor highlights the gravity of the anthrax situation in Ghana and advocates for comprehensive interventions using a One Health approach. The epidemiology of anthrax, including its historical roots and modes of transmission, is discussed. The consequences of anthrax outbreaks, such as severe illness, economic losses, and social distress, are outlined. To combat this complex issue, the letter emphasizes the importance of enhanced awareness, prevention, accurate diagnosis, and timely treatment. Recommendations include vaccination of animals and humans, education campaigns, proper disposal of infected carcasses, strengthening healthcare systems, surveillance, and early detection. Collaboration and coordination among professionals in the human, animal, and environmental sectors are crucial. By adopting a One Health approach and implementing these measures, Ghana can effectively mitigate the impact of anthrax outbreaks and safeguard the health and well‐being of its population and livestock.

## 1 INTRODUCTION

Anthrax, an infectious disease caused by spore‐forming bacteria known as *Bacillus anthracis*, manifests in various clinical forms, ranging from mild to severe presentations.^1^ The most common form is cutaneous anthrax, characterized by group of small blisters or bumps, itchy skin bumps, swelling, fever, vomiting, headaches, and muscle aches. Transmission typically occurs through exposure of cuts or abrasions to anthrax spores. Another form, gastrointestinal anthrax, is contracted by consuming meat from infected animals, initially resembling symptoms of food poisoning but progressing to haematemesis, severe abdominal pain, and intense diarrhea. The most severe and rarest variant is inhalation or pulmonary anthrax, which occurs when individuals directly inhale airborne anthrax spores. Recently, in northern Europe another type of anthrax infection is seen in people who use heroin through injections. This form of infection has similar symptoms to cutaneous form but infection may be deep inside the dermis.^2^ In anthrax infection, symptoms resemble those of a common cold, but they can rapidly escalate to severe respiratory distress and shock.^1^ Anthrax is prevalent in agricultural areas spanning Central and South America, sub‐Saharan Africa, central and southwestern Asia, southern and eastern Europe, as well as the Caribbean.^3^ Approximately 2000 cases of anthrax in humans occur each year worldwide.^4^ Ghana, situated in western Africa, has experienced a significant mortality rate associated with anthrax cases. Between 1980 and 2000, approximately 1000 individuals lost their lives to anthrax in Ghana.^5^ Recent studies examining the geographical distribution of anthrax risk in Ghana have supported these findings and have identified specific regions at high risk, namely Northern, Savannah, Upper West, North East, and Upper East regions.^6^ According to the Upper East Regional Director of the Ghana Health Service, around June 1, 2023, there has been a confirmed fatality and suspected anthrax infections among 11 individuals who consumed the carcass of dead cattle. On June 1, 2023, the Regional Health Directorate received a report of two anthrax cases, including one death, in the Binduri District, resulting from the consumption of dead cattle.^7^

In addition to the geographical factors, certain behaviors and practices have been identified that contribute to an increased likelihood of anthrax infection in livestock. These include neglecting livestock vaccination, despite the availability of locally produced vaccines, as well as engaging in activities such as slaughtering, butchering, and distributing the meat from animals that were either sick or had succumbed to anthrax.^5^ Addressing the anthrax threat requires a comprehensive approach that includes not only preventive measures but also effective treatment options. Fortunately, anthrax in humans responds well to antibiotics, particularly early in the course of the infection can significantly improve outcomes and reduce mortality rates associated with the disease. However, it is crucial to note that treatment efficacy may vary depending on the specific form and severity of the infection. Therefore, ensuring timely access to accurate diagnosis and appropriate medical care is essential in mitigating the impact of anthrax outbreaks. Doxycycline, ciprofloxacin, levofloxacin, and parenteral procaine penicillin G are the four Food and Drug Administration‐approved antibiotics for postexposure prophylaxis following exposure to *B. anthracis* spores which can be prescribed by the healthcare providers.^8^

The recent rise in anthrax infections among humans in Ghana has become a pressing public health concern, despite advancements in medical knowledge and preventive measures. This problem poses a continuous threat to human health and livelihoods, demanding immediate action. Anthrax not only jeopardizes human health but also undermines the socioeconomic fabric of Ghana. The agricultural sector, vital to the country's economy, suffers substantial losses due to anthrax outbreaks in livestock. The high livestock mortality rate associated with anthrax cases underscores the urgent need for enhanced awareness, prevention, and treatment strategies. By addressing the challenges posed by anthrax through a One Health approach, integrating human health, animal health, and environmental considerations, we can effectively combat this complex issue. This letter aims to raise awareness among policymakers, healthcare professionals, veterinary authorities, and the general public about the gravity of the anthrax situation in Ghana. Furthermore, it seeks to provide recommendations and advocate for comprehensive interventions encompassing prevention, early detection, accurate diagnosis, appropriate treatment, and education campaigns. By implementing these measures, we can significantly reduce the incidence of anthrax, mitigate its consequences, and safeguard the health and well‐being of the Ghanaian population and its livestock.

## 2 EPIDEMIOLOGY OF ANTHRAX

Since ancient times (700 BC), the disease anthrax has been well known. As far as is known, this disease is believed to have its roots in sub‐Saharan Africa, particularly in Egypt and Mesopotamia. The Iliad by Homer, written approximately 700 BC, and the verses of Virgil (who lived from 70 to 19 BC), provide evidence of anthrax in ancient Greece and Rome, where it is believed to have been spread globally. Maret and Fournier provided the first clinical descriptions of anthrax (cutaneous type); before their clinical representation, anthrax had only been documented historically.^9^ Anthrax was said to be responsible for both domestic and wild animals' greatest case mortality rates in the late 19th and early 20th centuries.^10^

Anthrax has been linked to a variety of hosts and environmental sources, including soil, fodder, bone meal, infected excreta, blood, and other discharges from diseased animals. The initial source of anthrax typically appeared when the soils of old anthrax burials were disturbed.^11^ Through contaminated waterways, insects, and the feces of sick birds and feral animals like cats, dogs, and other carnivores, the bacilli spread throughout the region. Concentrates, fodder, or contaminated animal products, such as bone meal, hides, hair, wool, fertilizers, and so forth, spread the virus into a new area.^11^

By ingestion, inhalation, or penetration through damaged skin, the bacilli enter the body. The primary method of infection in the majority of animal cases is ingestion of concentrate, forage, or water tainted with anthrax bacilli.^11^, ^12^ The entry of the bacilli into the system is facilitated by any wound in the mucous lining of the digestive tract. Although the inhalation infection (caused by contaminated dust) is always taken into consideration, it has little significance in animals. The most prevalent way for those working in associated industries to contract anthrax was through contact with contaminated hair, wool, hides, and skins.^11^, ^12^

In various anthrax epidemics in livestock and wildlife in India, Africa, and the United States, both biting (*Stomoxys* or *Tabanus* species) and nonbiting (blowfly and chrysoma species) insects have been implicated.^13^, ^14^ This mechanical mechanism of transmission causes edema after infection, which develops into the development of painless black lesions on the skin.^11^

## 3 OLD AND CURRENT CASES OF ANTHRAX IN GHANA

Anthrax is one of the earliest diseases of man and animal, it is cause by a bacterium called *B. anthracis*. This zoonosis is a major disease of public health concern. It is common in places where they have limited resources, underserved communities, rural agricultural areas as well as West Africa.^15^

Ghana has a history of human anthrax associated with a high case fatality rate according to study reports way back in 1980s, it says that nearly 1000 person died from anthrax.^5^ Death occurred as a result of spillover from infected livestock most especially in Northern Ghana.

On June 12, The Federal Ministry of Agriculture and Rural Development made it known to the general public on the Recent Outbreak of Anthrax in some neighboring countries within the West African Region, they include Northern Ghana, bordering Burkina Faso and Togo. Officials with the Ghana Health Service report a suspected anthrax outbreak in the Binduri District of the Upper East Region.

Human anthrax cases in the Upper East area were reported by district, with a single district in the Northern region reporting a single case. Upper East statistics were limited to aggregated annual counts per district from 2005 to 2015, with limited line list data available for 2016. In the Upper East, case‐patient age and gender were available for a subset of years from 2008 to 2014; other locations included human anthrax fatalities by month and year from 2005 to 2016. To compare to livestock reports, deaths were aggregated by month and year.

Between 2005 and 2016, a total of 38 anthrax‐related deaths were reported in Ghana, 30 in the Upper East and 8 in the Northern areas. Deaths were recorded in March, April, June, and December in eight cases with documented months of occurrence. Four deaths occurred in March and April, coinciding to seasonal peaks and regional concentrations of livestock anthrax.^6^, ^16^

Data from 83 human anthrax cases in the Upper East, including one area, West Mamprusi (now part of North East), revealed 30 (36.1%) deaths. Between 2005 and 2016, Bawku West had four outbreaks, Talensi Nabdam had three, and other districts had one. Human anthrax cases peaked in 2006, 2008, and 2014.

In Upper East data with information on the age and gender of case patients, 75.6% (31/41) were men, which was similar with rates in a prior report.^17^ Men died at a rate of 48.4% (15/31) compared to 40.0% (4/10) of women. The median age for men was 38 years (range: 7–81 years) and 39.5 years (range: 4–61 years) for women in all instances having age data.

## 4 CONSEQUENCES OF ANTHRAX IN GHANA

Anthrax outbreaks in Ghana have wide‐ranging consequences encompassing human health, the agricultural sector, and social well‐being. In terms of human health, anthrax infections can result in severe illness, long‐term disabilities, and fatalities, as highlighted by recent cases, including the incident in Binduri District. Effective preventive measures, accurate diagnosis, and timely treatment are urgently needed to mitigate the health consequences. The agricultural sector, crucial to Ghana's economy, bears the brunt of anthrax outbreaks, leading to significant economic losses due to the susceptibility of livestock, such as cattle, sheep, and goats. Sudden deaths of infected animals impose substantial financial burdens on farmers and livestock owners. Furthermore, the fear of anthrax contamination disrupts trade and export of livestock and their products, impeding economic growth and adversely affecting livelihoods. Anthrax outbreaks also have profound social and psychological impacts, instilling fear, vulnerability, and distress within communities. Stigmatization, isolation, and social unrest may arise, exacerbating the psychological burden on individuals and families. Moreover, the strain on healthcare systems and limited resources dedicated to managing anthrax cases further strain the overall health infrastructure, hindering the provision of essential healthcare services to the population. These comprehensive strategies may protect and safeguard human health, prevent economic losses, and enhance the social well‐being of affected communities.

## 5 ONE HEALTH TO MITIGATE ANTHRAX

One Health approach recognizes that human health is dependent on the health of animals, plants, and their shared environment.^14^ It emphasizes communication, coordination, and collaboration among professionals in the human, animal, and environmental sectors, as well as other relevant disciplines to mobilize resources and implement control measures to control diseases.^14^ Although it is very difficult to eradicate anthrax owing to the persistence of anthrax bacterial spores in the environment, it can be prevented,^18^ which can only be achieved using the holistic framework of the One Health approach for the prevention, detection, and effective response to the disease outbreak.^19^

To prevent zoonotic diseases, including anthrax, the One Health approach advocates for vaccination of animals to help prevent the spread of the disease to humans and protect individuals at high risk.^19^ Second, education and awareness campaigns are very important to inform communities about the risk, transmission pathways, and preventive measures to curb anthrax, which includes proper disposal of infected carcass animals.^19^ The One Health concept encourages a fast coordinated response that includes constant surveillance and early detection of the disease, timely treatment, and proper management of infected people and livestock, without neglecting the environment for the control and prevention of anthrax.^19^, ^20^ The success of the One Health program implemented towards anthrax control in Ghana include; ensuring a multidisciplinary and interdisciplinary collaboration among the physicians, nurses, pharmacists, veterinarians, including animal scientists and social workers in Ghana in mitigating anthrax outbreak in Ghana, ensuring animal vaccination, prompt diagnosis and treatment of the victims and limiting complications that could arise from the disease. It would also provide one health job opportunities, diversities among the health workers in Ghana and improve the economic development of Ghana as a country.

## 6 CONCLUSION AND RECOMMENDATIONS

The recent surge in anthrax infections in Ghana underscores the urgent need for comprehensive interventions to address this pressing public health concern. Anthrax poses a continuous threat to human health and the socioeconomic fabric of Ghana, particularly its agricultural sector. To effectively combat this complex issue, a One Health approach that integrates human health, animal health, and environmental considerations is paramount.

First and foremost, there is a critical need for enhanced awareness and education campaigns targeting Ghanaian policymakers, healthcare professionals, veterinary authorities, and the general public. These campaigns should emphasize the gravity of the anthrax situation in Ghana, raise awareness about transmission pathways, and promote preventive measures. Proper disposal of infected carcasses and vaccination of animals should be prioritized to prevent the spread of the disease and protect those at high risk. Timely access to accurate diagnosis and appropriate medical care is crucial in mitigating the impact of anthrax outbreaks. Efforts should be made to ensure that Ghanaian healthcare systems are equipped with the necessary resources and trained personnel to effectively manage anthrax cases. Strengthening surveillance systems and early detection mechanisms can facilitate prompt intervention and prevent further transmission.

Furthermore, collaboration and coordination among professionals in the human, animal, and environmental sectors are essential for a cohesive and efficient response. This includes constant surveillance, rapid reporting of cases, and prompt implementation of control measures. By adopting a One Health approach, Ghana can leverage the expertize and resources across disciplines to effectively combat anthrax and other zoonotic diseases. Additionally, research and monitoring efforts should be intensified to better understand the epidemiology and risk factors associated with anthrax in Ghana. This knowledge can inform targeted interventions and the development of more effective preventive strategies.

In conclusion, addressing the anthrax threat in Ghana requires a multifaceted approach that encompasses prevention, early detection, accurate diagnosis, appropriate treatment, and education campaigns. By implementing these recommendations and embracing the principles of One Health, Ghana can significantly reduce the incidence of anthrax, mitigate its consequences, and safeguard the health and well‐being of its population and livestock. Stakeholders at all levels of Ghana must come together, prioritize resources, and take decisive action to combat this persistent public health challenge.

## AUTHOR CONTRIBUTIONS

**Malik O. Oduoye**: Conceptualization; writing—original draft; writing—review and editing. **Godfred Y. Scott**: Conceptualization; writing—original draft; writing—review and editing. **Tirth Dave**: Project administration; supervision; writing—original draft; writing—review and editing. **Akanbi‐Hakeem H. Bolanle**: Writing—original draft; writing—review and editing. **Alexandra D. Mwinbong**: Writing—original draft; writing—review and editing. **Olajide O. Modupeoluwa**: Writing—original draft; writing—review and editing.

## CONFLICT OF INTEREST STATEMENT

The authors declare no conflicts of interest.

## TRANSPARENCY STATEMENT

The lead author Tirth Dave affirms that this manuscript is an honest, accurate, and transparent account of the study being reported; that no important aspects of the study have been omitted; and that any discrepancies from the study as planned (and, if relevant, registered) have been explained.

## Data Availability

The data used in this study are publicly available from the National Library of Medicine https://www.ncbi.nlm.nih.gov/. All relevant data are included in the manuscript. Additional data related to this study may be requested from the corresponding author upon reasonable request.
